# Self-care practices and support needs for breathlessness among outpatients with lung cancer: A qualitative study including family caregiver perspectives

**DOI:** 10.1016/j.apjon.2026.101018

**Published:** 2026-08-11

**Authors:** Ayumi Sugimura, Kikue Ushiyama, Shigeyoshi Maki, Shoko Ando

**Affiliations:** a Department of Nursing, Graduate School of Medicine, Gifu University, Gifu, Japan; b Department of Nursing, Japanese Red Cross Aichi Medical Center Nagoya Daiichi Hospital, Nagoya, Aichi, Japan; c Department of Integrated Health Sciences, Graduate School of Medicine, Nagoya University, Nagoya, Aichi, Japan; d Faculty of Nursing, Ichinomiya Kenshin University, Ichinomiya, Aichi, Japan

**Keywords:** Breathlessness, Caregivers, Dyspnea, Lung neoplasms, Qualitative research, Self-care

## Abstract

**Objective:**

This study aimed to explore self-care practices and support needs among outpatients with lung cancer experiencing breathlessness, with attention to family caregivers’ perspectives.

**Methods:**

This qualitative descriptive study used semistructured interviews with 13 outpatients with lung cancer experiencing breathlessness and 8 family caregivers, who were purposively recruited from respiratory outpatient clinics at two institutions. The 21 participants were interviewed in 15 sessions: seven patient-only, two family caregiver-only, and six joint patient–family caregiver sessions. Patient interviews were the primary data source, while family caregiver interviews were used as supplementary data rather than for dyadic comparison. Data were analyzed using qualitative content analysis based on Krippendorff's approach.

**Results:**

Patients’ self-care practices included encouraging oneself through successful coping experiences, monitoring and understanding breathlessness, using social support in daily life, performing direct care to relieve breathlessness, adjusting activity patterns to prevent or manage breathlessness, and preserving psychological stability. Their support needs included practical support for managing breathlessness, positive goal-setting that supports treatment continuation, a desire for breathlessness-related distress to be understood, coordination of support systems for home care, and explanations and advice for understanding breathlessness. Supplementary family caregiver data described the care they provided to patients, as well as their own needs for explanations, symptom-assessment guidance, and consultation and support resources.

**Conclusions:**

Outpatients with lung cancer practiced multidimensional self-care to manage breathlessness in daily life. These findings may inform family-inclusive outpatient breathlessness support that builds on patients' self-care practices, recognizes the care provided by family caregivers, and addresses family caregivers’ own support needs.

## Introduction

Breathlessness is a common and distressing symptom among patients with lung cancer and can substantially disrupt daily life. It is a complex physical and emotional symptom that patients may describe in terms of difficulty getting air in, chest tightness, or fear of dying.^1^, ^2^, ^3^, ^4^, ^5^ Breathlessness also restricts many aspects of daily living, including housework, mobility, and social activities.^1^^,^^2^^,^^4^^,^^6^

The importance of multidimensional support for breathlessness in patients with cancer, including both pharmacological and nonpharmacological approaches, has been increasingly recognized. For instance, the 2023 Japanese Society for Palliative Medicine guidelines recommend multidimensional assessment and support for breathlessness in patients with progressive disease, emphasizing nonpharmacological interventions, such as breathing techniques, behavioral adjustment, and environmental modification, as part of supportive care.^7^^,^^8^ Similarly, the American Society of Clinical Oncology (ASCO) guidelines recommend comprehensive management of breathlessness in advanced cancer, including assessing reversible causes and implementing nonpharmacological interventions.^9^ According to a systematic review, nonpharmacological interventions such as fan therapy, pulmonary rehabilitation, and multicomponent interventions may lead to improvements in breathlessness.^10^ Other systematic reviews also suggested that airflow directed to the face may help relieve breathlessness.^11^^,^^12^

However, these approaches have largely been presented as recommended interventions or symptom-management strategies, whereas less is known about how outpatients with lung cancer adapt, combine, and modify these approaches as part of their everyday self-care while continuing treatment. Breathlessness may also occur as episodic breathlessness, whose characteristics and conceptualization have been increasingly discussed.^13^^,^^14^ Patients experiencing episodic breathlessness use various self-management strategies, including activity adjustment, breathing techniques, cognitive strategies, and environmental modification.^15^ Nevertheless, existing evidence has not sufficiently clarified how patients interpret breathlessness, monitor symptom changes, decide when and how to respond, and incorporate these strategies into their daily routines in outpatient settings, where immediate access to health care professionals is often limited. To develop feasible outpatient nursing support that builds on patients’ existing self-care, nurses need to understand how patients apply, adapt, and integrate breathlessness-related coping strategies into daily life.

Family caregivers who support patients with breathlessness also play an important role in home care. These family caregivers express a desire to learn about breathlessness, appropriate responses to worsening symptoms, proper anxiety and panic management, and what to expect from this condition; they have also reported unmet needs regarding how to support patients experiencing such symptoms.^16^^,^^17^ Furthermore, family caregivers of patients with episodic breathlessness reportedly develop their own supportive strategies while experiencing anxiety and helplessness.^18^ However, in Japan, the kinds of care family caregivers provide and the type of support they need in the context of outpatient cancer care remain largely unknown.

Family caregivers' perspectives can further illuminate how patients’ breathlessness self-care is supported in everyday home settings. Family caregivers typically assist with symptom monitoring, activity adjustment, clinic attendance, and decisions about when to seek professional help. Clarifying their involvement and support needs is clinically important because outpatient breathlessness support often needs to be implemented at home, where patients and family caregivers manage symptoms together. Therefore, although patient interviews constituted the primary data source in this study, examining the care provided by family caregivers and their own support needs as supplementary contextual findings may provide a more practical understanding of breathlessness self-care among outpatients with lung cancer and offer useful insights into family-inclusive outpatient oncology nursing support.

This study aimed to explore the self-care practices of outpatients with lung cancer experiencing breathlessness and their needs for professional support. It also explored the care provided by family caregivers for patients' breathlessness and family caregivers’ own support needs as supplementary contextual findings to inform family-inclusive support for home-based breathlessness management among these outpatients.

## Methods

### Study design and participants

In this qualitative descriptive study, semistructured interviews were conducted with outpatients with lung cancer who experienced breathlessness and with a subset of their family caregivers. Patient interviews constituted the primary data source. Family caregiver interviews were conducted not to facilitate dyadic comparisons between patients and family caregivers but to gather supplementary contextual data on home-based breathlessness management and to identify implications for family-inclusive outpatient breathlessness support.

### Participants

Patients were included if they met the following criteria: were aged at least 18 years, attended the respiratory outpatient clinics of the collaborating institutions, had been diagnosed with stage III or IV lung cancer, had exertional breathlessness with a visual analog scale score of two or higher, and were capable of participating in an interview without excessive physical burden according to their attending physician's judgment. Conversely, the exclusion criteria were the presence of a psychiatric disorder, eligibility for surgical treatment, and transient worsening of breathlessness caused by infection or other acute conditions, as judged by the attending physician.

Eligibility criteria, including lung cancer diagnosis, stage III or IV disease, exertional breathlessness with a visual analog scale (VAS) score of two or higher, and ability to participate in an interview without excessive physical burden, were confirmed by collaborators at each institution based on medical records and clinical judgment. The participant characteristics shown in Table 1 were recorded from the research interview data sheet. Resting and exertional breathlessness intensity were assessed using a visual analog scale ranging from 0 to 10, with 0 indicating no breathlessness and 10 indicating the worst imaginable breathlessness. These VAS scores were self-reported by participants and recorded on the research interview data sheet at the time of the research interview. Performance status was assessed using the Eastern Cooperative Oncology Group performance status scale and is reported as Eastern Cooperative Oncology Group performance status (ECOG PS).

**Table 1:** Participant characteristics.

	Patient characteristics									Family caregiver characteristics			
Participant ID	Age (years)	Sex	Time since diagnosis	Resting VAS	Exertional VAS	ECOG PS	Current treatment	Home oxygen therapy	Cohabiting family members	Age (years)	Sex	Relationship	Employment
1	70s	Female	7 months	3	10	3	Pharmacotherapy	No	Daughter/grandchild				
2	70s	Male	1 month	2.5	3.5	2	Pharmacotherapy	Yes	None	60s	Male	Younger brother	Employed
3	60s	Male	2 years	2.5	3.5	2	Pharmacotherapy	No	Wife				
4	60s	Female	7 years	0	7.5	1	Pharmacotherapy	No	Mother	60s	Female	Older sister	Employed
5	70s	Male	5 years	0	8	0	Pharmacotherapy	No	Wife				
6	70s	Male	10 years	0	5	1	Symptom palliation	No	Wife	70s	Female	Wife	Not employed
7	60s	Male	5 months	0	2	0	Radiotherapy	No	None				
8	70s	Male	18 months	6.5	8	2	Symptom palliation	Yes	Wife	60s	Female	Wife	On caregiving leave
9	50s	Male	23 months	0	3	1	Pharmacotherapy	No	Wife/son				
10	70s	Female	15 months	0	5	2	Pharmacotherapy	No	Husband/son	70s	Male	Husband	Not employed
11	80s	Male	3 months	0	Not recorded	1	Pharmacotherapy	No	Wife/son and daughter-in-law	80s	Female	Wife	Not employed
12	70s	Male	12 months	0	3	2	Symptom palliation	Yes	Daughter/grandchild	50s	Female	Daughter	Employed
13	70s	Female	2 months	0	2	1	Pharmacotherapy	No	Husband	70s	Male	Husband	Not employed

Patient and family caregiver characteristics are linked by the same participant ID. Family characteristics are shown only for participants whose family caregivers were interviewed. ID, participant identification number; ECOG PS, Eastern Cooperative Oncology Group performance status; VAS, visual analog scale. Breathlessness VAS scores range from 0 to 10, with 0 indicating no breathlessness and 10 indicating the worst imaginable breathlessness. Resting and exertional breathlessness VAS scores were self-reported by participants and recorded on the research interview data sheet at the time of the research interview. For Participant 11, exertional breathlessness with a VAS score ≥ 2 was documented in the physician's medical record at recruitment. However, an exact current exertional VAS value was not obtained during the research interview; therefore, this value was recorded as “Not recorded” in the research data sheet and reported as “Not recorded” in Table 1.

Eligible family caregivers were adults aged 18 years or older who were identified by participating patients as their primary caregiver.

### Recruitment

Patients who met the eligibility criteria were identified and referred by attending physicians at the respiratory outpatient clinics of the two collaborating institutions. We provided oral and written explanations of the study to the referred patients and enrolled those who gave written informed consent. After acquiring patient consent, we invited the primary family caregivers to participate and obtained their written informed consent. This recruitment strategy was purposive, selecting participants according to predefined clinical criteria relevant to the study aim, namely, patients with outpatient lung cancer experiencing exertional breathlessness and family caregivers involved in their daily care. This recruitment approach was also constrained by clinical feasibility because eligible patients needed to be physically able to tolerate the interview without excessive burden.

Potentially eligible patients were initially identified by attending physicians and collaborators at each outpatient clinic according to the eligibility criteria and their physical ability to participate in an interview. The research team approached only those patients who were referred by the attending physicians and collaborators. All 13 referred patients agreed to participate and were therefore included in the analysis. Given this recruitment process, the total number of patients and family caregivers who were clinically eligible but not referred to the research team was not fully recorded. Family caregivers were invited after the participating patient had provided consent and when the primary family caregiver was available and willing to receive information about the study. For the remaining four patients, family caregivers were not approached because the patients reported that their family members were unable to participate owing to work commitments or living far away. Of the nine family caregivers who received an explanation of the study, eight agreed to participate, whereas one declined.

### Data collection

Data were collected between June 2023 and March 2026 through semistructured interviews with patients and their family caregivers. Recruitment was conducted from January 25, 2023 to March 31, 2024, at the first collaborating institution and from January 17, 2024 to March 31, 2026, at the second collaborating institution. Data collection was prolonged because external researchers were restricted from accessing the outpatient areas of the initial institution even after the COVID-19 pandemic, causing delayed and limited recruitment and interview opportunities. Given that only a few eligible patients could be recruited at the first institution, recruitment at that institution was discontinued on March 31, 2024, and an additional collaborating institution was secured to continue participant recruitment.

Recruitment and data collection were conducted under the approved study procedures throughout the study period, except that no participant-facing research activities were conducted between April 1, 2025 and June 4, 2025 during the first author's institutional transfer and additional ethics review at Gifu University.

Initially, patient and family caregiver interviews were planned as separate interviews. However, many patients wished to be interviewed together with their family caregivers, given that they received daily support from them. Therefore, interviews were conducted either separately or jointly according to participants' preferences. During analysis, the research team assessed whether differences in recruitment site, data collection period, or interview format affected participants’ accounts. Subsequently, no major differences requiring separate site-level analysis were identified.

The interviews were conducted in the collaborating institutions by the first author, who had experience in oncology nursing and qualitative research, and were audio-recorded with participants' consent. A total of 21 participants were interviewed, comprising 13 patients and 8 family caregivers. These interviews were conducted in 15 interview sessions: seven patient-only sessions, two family caregiver-only sessions, and six joint patient–family caregiver sessions. The seven patient-only sessions included five sessions with patients whose family caregivers did not participate and two separately conducted patient sessions from patient–family caregiver pairs. The two family caregiver-only sessions corresponded to the separately conducted family caregiver sessions from these pairs. The six joint sessions were conducted with both the patient and family caregiver present according to participants’ preferences. The reported interview duration refers to each interview session. Interview sessions lasted approximately 33 min on average, with a range of 13–45 min. Given that participating patients experienced breathlessness, interviews were conducted flexibly according to their physical status to minimize burden.

Patients were asked about the impact of breathlessness on daily life, the strategies they used to cope with it, and the support they sought from health care professionals. For family caregivers, the author asked about their experiences of supporting patients with breathlessness, the care they provided, and the support they sought from health care professionals. Supplementary Table S1 lists representative interview questions. Prompts were used as needed to elicit concrete examples, including changes in daily or social activities, psychological effects, specific strategies or care practices, and support received or desired during hospitalization or outpatient care. The interview guides were not pilot-tested and were not modified during the study period. The first author conducted all interviews, and no repeat interviews were performed.

### Data adequacy

Data adequacy was considered using the concept of information power, as proposed by Malterud et al., instead of full data saturation.^19^ Rather than being used as a prospective recruitment rule, information power was utilized primarily as a retrospective framework for assessing the sufficiency of the information provided by the final sample to address the study aim. The following factors were considered: the specificity of the study aim, the clinical specificity of the participant sample, the quality and relevance of the interview data, the exploratory descriptive analytic strategy, and existing knowledge regarding breathlessness and nonpharmacological symptom-management strategies. The study aim was focused on breathlessness self-care strategies and support needs among outpatients with lung cancer, and predefined clinical criteria relevant to this aim were employed to select patient participants. The interviews provided rich accounts of how breathlessness affected patients’ daily lives, how they monitored and managed symptoms, and what professional support they needed. While existing knowledge on breathlessness management and nonpharmacological symptom-management strategies informed the study background and interview topics, prior evidence specifically addressing breathlessness self-care and support needs among outpatients with lung cancer, including supplementary family caregiver perspectives, remains limited.

Family caregiver interviews were intended to provide supplementary contextual information rather than serve as a parallel dyadic dataset; therefore, data adequacy was considered separately for the patient and family caregiver datasets. Preliminary patient categories had been developed after the first 12 patient interviews. To further confirm the adequacy of these categories, we conducted one additional patient interview. This additional interview did not generate new categories or subcategories but provided confirmatory data within the existing category structure. For the family caregiver dataset, the interviews provided supplementary information regarding home care and family caregivers' own support needs. However, because this dataset was supplementary and smaller in size, it provided contextual rather than comprehensive coverage of family caregivers’ perspectives.

After reviewing the available interviews, the development of categories, and the confirmatory role of the additional patient interview, the research team judged that the data were adequate to identify the key aspects of patients' breathlessness self-care and support needs as well as family caregivers’ supportive actions and support needs in this outpatient context. Therefore, additional interviews were not pursued.

### Data analysis

Qualitative content analysis based on Krippendorff's approach was used for data analysis.^20^ Patient data were analyzed to clarify the self-care practices and support needs of outpatients with lung cancer experiencing breathlessness. For the patient analysis, participant-level patient data constituted the sampling unit. In patient-only sessions, the transcript of that session was used as the source document. In joint interview sessions, one speaker-labeled transcript was produced for each session, and patient utterances were extracted and entered into the patient analysis sheet. The recording unit was one meaning unit representing either a self-care practice for breathlessness or a support need related to breathlessness. The context unit was the participant-level patient data, including the relevant interviewer utterances and surrounding interactional context.

Meaning units were identified as the smallest segments of text that carried a coherent central meaning, rather than being divided by sentence or grammatical structure. The analysis focused on participants’ utterances concerning their practical strategies and adjustments used to cope with breathlessness, as well as their needs for explanations, understanding, emotional support, and support in daily living. Narratives describing how breathlessness affected daily life were used as contextual information and referenced as needed when interpreting meaning units.

The transcripts were organized into analysis sheets while preserving the original data. These sheets included participant ID, utterance sequence, speaker, verbatim text, immediately preceding interviewer utterance, candidate analytic content, broad classification, meaning unit, initial code, subcategory, category, and memo. In interviews conducted with both participants present, patient, family caregiver, and interviewer utterances were clearly labeled by speaker in the transcripts. Patient and family caregiver utterances were entered into separate analysis sheets and analyzed separately according to the analytic focus of each dataset. When one participant supplemented the other's account, the utterance was interpreted according to the speaker and interactional context. Data coding and categorization were primarily based on participants' utterances. The analytical process proceeded from the preparation of verbatim transcripts to the development of meaning units, initial codes, subcategories, and categories. For example, a patient's statement about anticipating possible physical changes during travel and subsequently adjusting transportation plans, rest, and scheduling was extracted as a meaning unit concerning activity adjustment to prevent or manage breathlessness. This meaning unit was coded as “adjusting activity plans in anticipation of physical changes,” grouped into the subcategory “Adjusting activity level and daily plans,” and then organized under the category “Adjusting activity patterns to prevent or manage breathlessness.”

The same analytic procedure was applied to the family caregiver transcripts, which were analyzed as supplementary data to the patient data. For the family caregiver analysis, participant-level family caregiver data constituted the sampling unit. In family caregiver-only sessions, the transcript of that session was used as the source document. In joint interview sessions, one speaker-labeled transcript was produced for each session, and family caregiver utterances were extracted and entered into the family caregiver analysis sheet. The recording unit was one meaning unit representing either breathlessness-related care provided by the family caregiver or a support need expressed by this family caregiver. The context unit was the participant-level family caregiver data, including the relevant interviewer utterances and surrounding interactional context. The analysis focused on family caregivers' utterances regarding the care, consideration, and support they actually provided in patients' daily life to address breathlessness, including assistance with clinic visits and transportation, strategies to prevent symptom worsening, and the explanations, criteria for judgment, practical advice, and outpatient support they sought from health care professionals. The categories related to family care and family caregivers’ support needs were not predetermined from the patient-derived categories; instead, they were developed from the family caregiver transcripts.

To enhance the trustworthiness of the analysis, the first author served as the primary analyst and repeatedly read all transcripts, extracted meaning units, and developed initial codes, subcategories, and categories while maintaining analytic memos. The preliminary coding and categorization results were reviewed by the research team, which comprised researchers with experience in oncology nursing, palliative care, and qualitative research. Rather than coding independently across all transcripts, the research team reviewed and discussed the analytic process and category development following the first author's initial analysis. In response to the team's comments and discussions, the first author returned to the original transcripts, re-examined the relevant meaning units, and revised the coding and categorization as needed. This iterative process was repeated until the team reached consensus on the final categories. An audit trail was maintained using analysis sheets that documented the progression from verbatim data to meaning units, initial codes, subcategories, categories, analytic memos, and records of team discussions. The reporting of this qualitative study was guided by the Consolidated Criteria for Reporting Qualitative Research.

Considering participants’ physical burden related to breathlessness and the practical difficulty of recontacting participants during outpatient treatment, we did not conduct member checking or participant validation. Instead, the trustworthiness of the analysis was supported through repeated comparison with the original transcripts, analytic memos, team discussions, and the audit trail.

All interviews were conducted and analyzed in Japanese. Quotations presented in this manuscript were translated into English by the first author after completing the analysis, and were subsequently checked through professional English language editing. The other authors then reviewed the translated quotations against the original Japanese statements and revised the wording where necessary to improve readability while preserving the intended meaning of the participants’ original words.

### Reflexivity

The interviews were conducted by the first author, a female nurse researcher who held a PhD at the time of data collection and had experience in oncology nursing, palliative care, and qualitative research. Field notes were made during or immediately after the interviews and were used to support interpretation during analysis. This first author was not affiliated with the two collaborating institutions and had no direct clinical care relationship with the participants; thus, participants would likely feel less constrained when describing their support needs or experiences with health care professionals. However, given the potential influence of the interviewer's professional background on the interpretation of participants' experiences of breathlessness and care needs, reflexive attention was maintained throughout data collection and analysis. The first author kept analytic memos documenting impressions, assumptions, and interpretive decisions and repeatedly returned to the original transcripts when developing and revising codes and categories. Interpretations were also discussed with the research team to ensure that the categories remained grounded in the participants' accounts.

## Results

### Participant characteristics

This study included 13 patients, comprising 9 male and 4 female. They were in their 50s–80s and had been diagnosed 1 month to 10 years earlier. Current treatment included pharmacotherapy, symptom palliation, and radiotherapy in 9, 3, and 1 participants, respectively. Home oxygen therapy had been introduced in 3 participants. Eight family caregivers, including wives, husbands, a daughter, and siblings, participated in family caregiver interviews. Of the 13 participating patients, three were recruited from the first institution and 10 from the second institution. Of the eight participating family caregivers, one was recruited from the first institution and seven from the second institution. Table 1 shows the participant characteristics.

### Self-care practices used by outpatients with lung cancer to manage breathlessness

Patients’ self-care practices were organized into six categories: encouraging oneself through successful coping experiences, monitoring and understanding breathlessness, using social support in daily life, performing direct care to relieve breathlessness, adjusting activity patterns to prevent or manage breathlessness, and preserving psychological stability (Table 2).

**Table 2:** Patients’ self-care practices for breathlessness.

Subcategory	Category
Gaining confidence through trying one's own coping strategies	Encouraging oneself through successful coping experiences
Urging oneself on despite breathlessness	
Drawing encouragement from others' affirmation of coping efforts	
Seeking explanations about the causes and treatment of breathlessness	Monitoring and understanding breathlessness
Considering how to respond when breathlessness worsens	
Self-monitoring and judging breathlessness	
Seeking help from family caregivers and others	Using social support in daily life
Using external resources to reduce daily burden	
Adjusting and using oxygen by oneself	Performing direct care to relieve breathlessness
Relieving breathlessness through cooling or airflow	
Modifying breathing patterns	
Adjusting activity level and daily plans	Adjusting activity patterns to prevent or manage breathlessness
Avoiding outings to reduce infection-related worsening	
Slowing movement pace	
Taking preventive breaks during activities	
Adjusting work or roles	
Resting after breathlessness occurs	
Finding ways to make eating easier	
Staying active to prevent physical decline	
Adjusting speech to avoid worsening breathlessness	
Managing environmental stimuli that trigger coughing	
Engaging in solitary distraction	Preserving psychological stability
Maintaining social connections to distract from breathlessness	
Calming anxiety or impatience	

Patients focused not only on relieving symptoms directly and adjusting their behavior but also on understanding changes in and meanings of breathlessness. They monitored their breathlessness status, oxygen saturation (SpO_2_), and pulse rate as cues for judging their lung condition and deciding how to respond. One patient mentioned using a commercially available pulse oximeter to check whether their pulse rate stabilized over time: “I bought a commercially available pulse oximeter. Sometimes, only my pulse is unusually high, even when my oxygen saturation is fine. If I wait for a while, it goes down, and that makes me feel calmer.” (P3)

To prevent or manage breathlessness through activity adjustment, patients slowed their pace, took breaks, adjusted their speech, managed environmental stimuli that triggered coughing, and minimized exertion. One patient described deliberately reducing walking speed to make ambulation more manageable: “If I walk slowly, even about 10 m is probably manageable. I walk at a pace that would seem slow even for an ordinary person.” (P8)

Furthermore, patients performed direct care, including oxygen use, breathing adjustment, cooling, airflow, and positioning, to relieve or cope with breathlessness. Some strategies were used both for relieving symptoms and for distracting attention from breathlessness. One patient described using cooling as a way to cope with breathlessness-related discomfort: “I put an ice pillow on my neck, wrapped in several towels. It may not actually make the breathlessness easier, but it distracts me. I try to take my mind off it because otherwise, I keep thinking only about how hard it is.” (P1)

Additionally, to help preserve psychological stability, some patients changed their surroundings and distracted themselves from breathlessness-related anxiety and discomfort. One patient described going outside to improve their mood when staying indoors increased feelings of fear and frustration: “I would feel overwhelmed if I did not distract myself. I would go outside, sit quietly in the shade, try to change my mood, and walk if I could.” (P3)

Patients also used social support to reduce movement burden and sustain daily activities despite breathlessness. One patient described relying on people around them when mobility became difficult: “It was difficult and felt unsafe, but people around me helped me move forward a little and waited for me. It was hard just to get to a seat, but their help made it manageable.” (P4)

Some patients encouraged themselves through successful coping experiences, such as engaging in activities they were still able to perform, gaining a sense of achievement, and drawing encouragement from others' affirmation of their coping efforts. One patient said, “When I think, ‘I went out today’ or ‘I took a walk today,’ I feel a sense of achievement, and that makes me feel good.” (P11)

Overall, self-care involved managing symptoms while maintaining daily functioning despite breathlessness.

### Support needs of outpatients with lung cancer from health care professionals

Patients’ support needs were organized into five categories: practical support for managing breathlessness, positive goal-setting that supports treatment continuation, a desire for breathlessness-related distress to be understood, coordination of support systems for home care, and explanations and advice for understanding breathlessness (Table 3).

**Table 3:** Patients’ support needs from health care professionals.

Subcategory	Category
Receiving practical guidance on specific methods to relieve breathlessness (e.g., comfortable positioning)	Practical support for managing breathlessness
Receiving guidance on breathing techniques and body movements	
Receiving assistance in judging symptom severity and coping thresholds	
Setting goals that foster hope and a sense of future outlook	Positive goal-setting that supports treatment continuation
Wanting suffering and activity limitations to be understood	A desire for breathlessness-related distress to be understood
Wanting responses that do not make asking for help feel futile	
Wanting supportive presence when distressed	
Wanting breathlessness not to be judged solely by health care professionals' assessment of symptom severity	
Expanding home-care services	Coordination of support systems for home care
Receiving care certification and support coordination appropriate to illness severity	
Receiving advice tailored to individual symptoms and side effects	Explanations and advice for understanding breathlessness
Receiving explanations about treatment, procedures, and future outlook	
Receiving explanations about how illness, treatment, and side effects affect breathlessness	

Patients sought practical support for managing breathlessness, including concrete guidance on breathing techniques and body movements in daily life. One patient described having to learn through trial and error how to move without worsening breathlessness: “I kept being told, ‘There is nothing that can be done. You just have to do rehabilitation by yourself.’ I thought, ‘So that is how it is.’ I could not even understand what it meant that I could not climb the stairs at home. Even now, if I forget and move too quickly, I feel breathless. Now I have learned what makes me breathless, so I try to move accordingly, but the symptoms themselves have not changed.” (P4)

Patients also wanted help from health care professionals with setting positive, near-future goals to support their motivation to continue treatment. One patient said, “If they could tell me about a small, bright goal ahead, I might feel that I could try a little harder.” (P3)

Patients also wanted individualized explanations about their condition's symptoms, treatment, and prognosis. In particular, some patients tried to understand why breathlessness persisted despite experiencing seemingly effective treatment. One patient described asking about the relationship between breathlessness and lung lesion status: “I asked the doctor today, ‘I feel breathless, but what is happening in my lungs?’ The doctor said the tumor had become smaller than before and seemed to be gradually shrinking. If that is the case, I wonder why I still feel breathless.” (P1)

Additionally, patients strongly desired that health care professionals understand the suffering and limitations associated with breathlessness and avoid judging symptom severity solely from a professional perspective. One patient described how a reassuring statement from health care professionals could make expressing current distress difficult: “If they say, ‘You have calmed down now, so you are all right, aren't you?’ I cannot say anything more.” (P3)

Some patients also wanted home-care services to be better coordinated and expanded so that practical support could be provided within the limited time available. One patient described how the limited duration of home-care services made it difficult to receive all of the assistance they needed: “When they come for 45 minutes, the time is already over after we talk for a while. Even though there are things I really want them to do, I end up thinking, ‘There is not enough time, so never mind.’” (P2)

Overall, patients needed practical guidance for managing breathlessness, explanations that helped them understand symptoms and treatment, positive goal-setting, coordination of home-care support, and responses that acknowledged the distress and uncertainty associated with breathlessness.

### Care provided by family caregivers

As supplementary contextual findings, care provided by family caregivers for patients was organized into six categories: psychological support among family members, information sharing among family members regarding the patient's condition, support for the patient's distraction and sense of purpose, direct support to ease breathing, support for maintaining the patient's daily life and activities, and coordination of medical visits and social resources to sustain treatment and care (Table 4).

**Table 4:** Care provided by family caregivers for patients’ breathlessness.

Subcategory	Category
Sharing anxiety and supporting one another among family members	Psychological support among family members
Sharing and monitoring information regarding the patient's condition among family members	Information sharing among family members regarding the patient's condition
Securing means of contact in preparation for emergencies	
Facilitating distraction and a sense of purpose in daily life	Support for the patient's distraction and sense of purpose
Practicing breathing techniques together with the patient	Direct support to ease breathing
Assisting in stretching and comfortable positioning	
Adjusting the living environment to minimize burden	Support for maintaining the patient's daily life and activities
Taking over household tasks	
Assisting with transportation and mobility during outings	
Watching over the patient's daily activities	
Arranging social insurance and care services	Coordination of medical visits and social resources to sustain treatment and care
Confirming the adequacy of available social insurance and care services	
Making adjustments to sustain clinic attendance	

Family caregivers provided psychological support by acknowledging emotional concerns among family members and reassuring family members who felt responsible for care. One family caregiver said, “My mother lived with her and seemed to feel that she might have to see her daughter pass away before her because of the illness. She also seemed to feel responsible for taking care of my sister and staying well herself. I told her, ‘You are not the only one who has to take care of her. We are here, and we will do this together. I live nearby, so if she really becomes unable to move, I can come and see her.’ I think my mother may have felt that she had to bear it all by herself.” (F4)

Family caregivers also shared information about the patient's condition with other family members to avoid placing excessive demands on the patient and to help them recognize changes in the patient's condition. One family caregiver said, “I tell my mother that treatment can cause breathlessness, fever, and other symptoms and that there may be times when she feels breathless. I ask her not to make unreasonable requests at such times. Because my mother is closest to her, I also tell her to call me whenever she is worried, so that I can check the situation.” (F4)

To support the patient's distraction and sense of purpose, family caregivers created opportunities for ordinary activities outside the home. One family caregiver said, “When I have a day off, I sometimes take him out for coffee.” (F12)

As direct support to ease breathing, family caregivers encouraged patients to breathe through the nose during oxygen use. One family caregiver said, “I tell her that mouth breathing does not work, so he should always breathe in through his nose.” (F12)

Family caregivers also supported patients' daily life and outpatient treatment by assisting with transportation, mobility, household tasks, and care coordination. One family caregiver described providing transportation, checking the patient's condition, and helping with shopping to support outpatient visits and daily life: “My role is mainly taking her to and from the hospital. I also ask whether she needs anything, and if she does, I buy it for her. I look at how she is doing and ask whether she wants to go shopping.” (F10)

To reduce the burden of continued outpatient treatment, family caregivers coordinated medical visits. One family caregiver reported arranging multiple consultations on the same day: “He sees respiratory medicine, urology, and orthopedics, and they see him at the same time, so he visits three departments in one day.” (F6)

Overall, family caregivers supported patients' breathlessness management not only by providing direct physical care but also by helping maintain patients’ daily life, attending outpatient appointments, and continuing treatment at home.

### Support needs of family caregivers

As supplementary contextual findings, family caregivers' own support needs were grouped into four categories: explanations for family caregivers regarding disease status and symptoms, guidance to support family caregivers’ judgment and management of breathlessness, accurate assessment of breathlessness severity in outpatient settings, and information on support resources for continuing home care (Table 5).

**Table 5:** Family caregivers’ support needs from health care professionals.

Subcategory	Category
Including family caregivers during explanations and consultation	Explanations for family caregivers regarding disease status and symptoms
Receiving individualized explanations beyond general symptom information	
Receiving explanations that convey symptom stability	
Receiving explanations about care methods needed for home care	Guidance to support family caregivers' judgment and management of breathlessness
Receiving explanations about criteria for judging worsening symptoms and when to respond	
Receiving suggestions for specific ways to manage breathlessness	
Assessing the impact of breathlessness on daily life at home	Accurate assessment of breathlessness severity in outpatient settings
Appropriately assessing breathlessness during limited outpatient visits	
Receiving information about consultation resources for home care	Information on support resources for continuing home care
Receiving information about support systems for sustaining long-term home care	

Family caregivers wanted understandable explanations about the patient's disease status and symptoms, including explanations that reassured them about symptom stability. Some family caregivers had difficulty understanding brief clinical explanations and wanted clear explanations tailored to nonprofessionals. One family caregiver said, “The doctor's explanations are a little too brief, and it is difficult for a layperson to understand. I wish the explanations were a little easier to understand. The doctor may understand by looking at the X-ray, but we cannot tell what is there or what is not just by looking at it.” (F10)

Family caregivers also desired practical guidance on how to judge worsening symptoms and how to respond at home. In particular, they wanted health care professionals to provide concrete examples of symptom changes prompting consultation. One family caregiver explained, “For example, I would like them to say something specific, such as, ‘If you used to be able to walk to the station but now become exhausted halfway, please let us know.’” (F4)

They also wanted a more accurate assessment of breathlessness, including its impact on daily life at home, within the limited time available during outpatient visits. Some family caregivers believed that measurements taken during outpatient visits did not necessarily reflect the breathlessness patients experienced during their daily activities at home. One family caregiver said, “They measure oxygen saturation during outpatient visits, but I sometimes think, ‘That is the saturation while the patient is sitting.’” (F4)

In addition to assessment and guidance, family caregivers sought access to consultation resources and information on support systems to sustain home care. One family caregiver described how the caregiving burden on the family was alleviated through informal community support: “There was someone who helped as a volunteer, not through long-term care insurance. They even helped take out the garbage. Because we had someone like that, we could rely on them to some extent. If we tried to do everything ourselves, we would probably become exhausted along the way.” (F2)

Overall, family caregivers needed understandable explanations, practical judgment criteria, accurate assessment of breathlessness in outpatient settings, and accessible consultation and support resources to manage patients’ breathlessness and continue home care with greater confidence.

## Discussion

This study explored the self-care practices and needs for professional support among outpatients with lung cancer experiencing breathlessness, as well as the care provided by family caregivers for patients and family caregivers' own support needs. Results showed that patients not only engaged in direct symptom relief and behavioral adjustment but also tried to understand changes in and meanings of their symptoms as part of self-care. Additionally, both patients and family caregivers expressed a need for health care professionals to recognize and adequately assess breathlessness and its associated distress, even when such distress was difficult to verbalize. Furthermore, patients’ nonpharmacological strategies included not only approaches consistent with recommended interventions (e.g., breathing techniques and behavioral adjustments) but also other coping strategies developed through daily experience.

First, notably, patients not only practiced direct symptom relief and behavioral adjustment but also exerted efforts to understand their own symptoms as part of self-care. Patients monitored their own SpO_2_ and subjective breathlessness and tried to comprehend the causes and meanings of their symptoms while deciding how to respond when breathlessness worsened. As such, understanding their symptoms not only enabled them to acquire knowledge but also facilitated decision-making in daily life. O'Driscoll et al. and Booth et al. reported that breathlessness resulted in wide-ranging restrictions on patients' lives and that many patients eventually developed their own ways of coping.^1^^,^^2^ Brown et al. also showed that patients with lung cancer used strategies such as activity adjustment and positive reframing to manage breathlessness.^4^ Moreover, Simon et al. reported that patients with advanced disease used various self-management strategies for episodic breathlessness.^15^ The present study adds to this literature by showing that trying to understand the meaning of symptoms may be an important component of self-care. Therefore, breathlessness support should not be limited to technical guidance, such as breathing techniques or activity adjustment; it should also include support for how patients interpret their symptoms and what cues they use in making decisions.

Second, patients' nonpharmacological coping strategies included both approaches overlapping with existing recommended interventions and other coping strategies developed through daily experience. To relieve breathlessness or prevent worsening, patients reported changing breathing patterns, taking rest, and adjusting activity level and pace. These strategies align with the concepts underlying nonpharmacological interventions described in the Japanese Society for Palliative Medicine and ASCO guidelines.^7^, ^8^, ^9^ Systematic reviews have also suggested that fan therapy and multicomponent nonpharmacological interventions can improve breathlessness.^10^ According to reviews focusing on fan therapy and airflow, airflow directed to the face helps relieve breathlessness; in a meta-analysis, the immediate effects of fan therapy were reported in patients with terminal cancer.^11^^,^^12^^,^^21^ However, patients in the present study not only applied facial airflow but also cooled their necks. Importantly, cooling was not always reported to directly relieve breathlessness; rather, it was sometimes used as a way to distract attention from breathlessness-related discomfort. These findings indicate that some patient-reported strategies overlapped with guideline-recommended nonpharmacological interventions, whereas others appeared to have evolved through daily experience. We interpret these practices as adaptations of coping strategies to patients’ own living environments, rather than as direct evidence that patients actively reconstructed or translated formal interventions. Therefore, future support should not merely provide standardized nonpharmacological interventions uniformly, but should also assess how patients already combine and adjust coping strategies in daily life and tailor guideline-supported advice to their individual activities, environments, and concerns.

Third, patients wanted to understand their symptoms and distress. Their support needs included wanting health care professionals to understand their suffering and activity limitations, to remain with them during distress, to avoid making them feel that seeking help was futile, and to respond without judging symptom severity solely from a health care professional perspective. These findings suggest a need extending beyond explanations about causes and treatment; in particular, patients wanted health care professionals to grasp their suffering and daily difficulties that were not always easily verbalized. Given that breathlessness itself can make speech difficult and that it may worsen during daily activities even when not apparent at rest, fully capturing it in the brief time available during outpatient visits may be difficult.^1^^,^^3^^,^^5^^,^^6^ O'Driscoll et al., Gysels et al., and Roberts et al. all demonstrated that breathlessness is a multidimensional experience involving physical, emotional, and social aspects, with discrepancies arising between patients' experiences and clinicians' perceptions.^1^^,^^3^^,^^5^ A qualitative study of advanced lung cancer also reported that episodic breathlessness was associated with substantial emotional distress.^22^ Therefore, these findings suggest that health care professionals should attend to changes in daily life and forms of suffering that are difficult to express, rather than relying only on explicit complaints or preconceived assumptions.

In the present study, supplementary family caregiver perspectives comprised two components: care provided by family caregivers for patients and family caregivers' own support needs. Family caregivers cared for patients by providing psychological support among family members, sharing information regarding the patients' condition, supporting patients' distraction and sense of purpose, directly supporting patients to ease breathing, offering support for daily life and activities, and coordinating medical visits and social resources. Family caregivers' own support needs included understandable explanations about patients' disease status and symptoms, guidance on how to judge worsening breathlessness and when to seek professional help, accurate outpatient assessment of breathlessness severity, and access to consultation and support resources. Farquhar et al. reported that caregivers of patients with breathlessness wanted to learn more about breathlessness, how to respond, and what to expect.^16^ Malik et al. found that caregivers of patients with breathlessness, including those with lung cancer, experienced substantial unmet needs and burden.^17^ Reitzel et al. further showed that while experiencing anxiety and helplessness, family caregivers of patients with episodic breathlessness developed coping strategies yet continued to have support needs.^18^ These two components complemented the patient findings by illustrating how patients’ breathlessness self-care was supported within everyday home contexts and highlighting the support that family caregivers themselves needed. Because the family caregiver data were analyzed as supplementary contextual data rather than as dyadic patient–family data, these findings should be interpreted as contextual insights rather than as evidence of concordance or discordance between patients and family caregivers. When considered together, however, the patient and supplementary family caregiver findings suggest two implications for outpatient breathlessness support. First, outpatient assessment should capture not only resting measurements but also activity-related breathlessness, daily-life limitations, and distress that patients may find difficult to verbalize, because patients wanted their suffering and activity limitations to be understood and family caregivers felt that outpatient measurements at rest did not necessarily reflect symptoms at home. Second, both patients and family caregivers were already engaged in practical management of breathlessness in daily life; patients used direct care and activity adjustment, whereas family caregivers provided direct support, daily-life assistance, and coordination of medical visits and social resources. However, both groups still needed guidance on symptom understanding, coping strategies, thresholds for consultation, and available support resources, suggesting the need for family-inclusive educational support.

Taken together, these findings suggest that outpatient support for breathlessness among outpatients with lung cancer may build on patients' multidimensional self-care practices while also addressing both patients' and family caregivers' needs for understanding and decision support. Future outpatient support for breathlessness should integrate direct guidance on nonpharmacological management with explanations that support understanding of symptoms, assessment of difficulties in daily life, and consultation and decision support involving both patients and family caregivers. Future work should examine how guideline-recommended nonpharmacological strategies can be adapted and evaluated in ways that are feasible for patients’ everyday lives.

These findings underscore that breathlessness support should be incorporated into outpatient oncology nursing practice. In Japan, patients with lung cancer often engage in time-limited outpatient consultations; consequently, breathlessness may be evaluated primarily as a symptom observed or reported during the consultation, rather than as a distressing experience that unfolds in everyday life. Participating patients experienced breathlessness while walking, eating, talking, attending clinic visits, and performing ordinary activities at home. Therefore, health care professionals need to assess breathlessness not only at rest but also in relation to daily activities, symptom fluctuations, self-care strategies, emotional distress, and situations wherein patients could not fully express their suffering.

Patients and family caregivers also need practical guidance and accessible resources to manage breathlessness outside the clinic, where they spend most of their time without immediate access to health care professionals. Thus, outpatient oncology and palliative care nurses may consider providing concrete, individualized guidance on feasible guideline-supported nonpharmacological strategies, including pacing, rest, breathing adjustment, positioning, and airflow directed to the face. They may also need to clearly explain signs of worsening breathlessness, thresholds for consultation, and available support pathways, including home care and palliative care resources. Despite brief outpatient encounters, acknowledging patients’ difficult-to-verbalize distress and offering practical management strategies may help patients and families feel better supported and better equipped to manage breathlessness in their daily lives.

### Limitations

This study has several limitations. First, it exclusively included outpatients with lung cancer and a subset of their family caregivers and had a small sample size; therefore, caution is needed when generalizing the findings. Second, family caregiver interviews were conducted with a subset of participants and were analyzed as supplementary contextual data. Therefore, this study did not conduct a full dyadic analysis of patient–family caregiver pairs, and family-related findings should be interpreted as contextual findings that complement the patient data. Third, given that this study included only patients who could participate in interviews without excessive physical burden, it may not have fully captured the experiences of patients with more severe or unstable breathlessness. This may have introduced selection bias and limited the transferability of the findings. Fourth, data collection was prolonged, partly because external researchers were restricted from accessing the outpatient areas of the first collaborating institution even after the pandemic, thereby delaying recruitment and interview opportunities. Although the eligibility criteria and interview guides remained unchanged, changes in outpatient care practices or family involvement during the extended data collection period may have influenced participants' accounts. While we considered differences in recruitment site, data collection period, and interview format during analysis, no major differences requiring separate site-level analysis were identified. Fifth, we did not conduct member checking; therefore, the derived categories were not returned to participants for validation. To address this limitation, we supported the analysis through repeated comparison with the original transcripts, analytic memos, team discussions, and the audit trail. Sixth, because most patient–family caregiver pairs were interviewed jointly according to participants’ preferences, the presence of the other family member may have influenced disclosure, particularly regarding caregiver burden, family tensions, unmet needs, and negative experiences.

## Conclusions

Outpatients with lung cancer experiencing breathlessness practiced multidimensional self-care that included direct symptom relief and behavioral adjustment, as well as efforts to understand their own symptoms and manage distress associated with breathlessness. Patients expressed a need for their difficult-to-verbalize breathlessness-related suffering to be understood. Supplementary contextual findings from family caregivers further showed that family caregivers provided practical support for breathlessness management at home while also requiring support for symptom understanding and decision-making. These findings may inform the future development and evaluation of outpatient breathlessness support that is grounded in patients’ existing self-care practices and complemented by family-inclusive explanations, decision support, and consultation and support resources.

## CRediT authorship contribution statement

Ayumi Sugimura: Conceptualization, Methodology, Investigation, Formal analysis, Data curation, Funding acquisition, Writing - original draft, Writing - review & editing. Kikue Ushiyama: Investigation, Validation, Writing - review & editing. Shigeyoshi Maki: Investigation, Validation, Writing - review & editing. Shoko Ando: Validation, Writing - review & editing. All authors approved the final manuscript.

## Ethics statement

The study was approved by the Ethics Review Committee of the Graduate School of Medicine, Nagoya University (Approval No. 2022-0193-4), and, following the first author's institutional transfer, by the Research Ethics Committee of the Graduate School of Medicine, Gifu University (Approval No. 2025-064). The required approval or permission was also obtained from each participating institution before data collection. No participant recruitment, informed consent procedures, interviews, or other participant-facing research activities were conducted between April 1 and June 4, 2025. The study was conducted in accordance with the Declaration of Helsinki and its subsequent amendments. Written informed consent was obtained from all participants before data collection.

## Data availability

The data that support the findings of this study are available from the corresponding author, AS, upon reasonable request and subject to applicable ethical and privacy restrictions. The data are not publicly available due to privacy and ethical restrictions, as they contain confidential de-identified interview data from patients and family caregivers.

## Declaration of generative AI and AI-assisted technologies in the writing process

During the preparation of this work, the authors used AI-assisted tools to support English language refinement and improve readability. The authors reviewed and edited all AI-assisted output and take full responsibility for the content of the published article.

## Funding

This study was supported by 10.13039/501100001691JSPS KAKENHI (Grant No. 22K17450). The funder had no role in the study design, data collection, analysis, interpretation of data, manuscript preparation, or decision to submit the article for publication.

## Declaration of competing interest

The authors declare there are no conflicts of interest.
