# Supplementary material for: Self-care practices and support needs for breathlessness among outpatients with lung cancer: A qualitative study including family caregiver perspectives
Source: Asia Pac J Oncol Nurs. 2026 Aug 11;13:101018. doi: 10.1016/j.apjon.2026.101018 (PMC13631346; doi:10.1016/j.apjon.2026.101018)
Supplement: Supplementary file 1 [file mmc1.docx]

**Supplementary Table S1. Representative interview questions**

| **Participant group** | **Representative questions and prompts** |
| --- | --- |
| Patients | How has breathlessness affected your daily life? |
|  | Are there any daily activities that you can no longer do or have changed the way you do because of breathlessness? Please describe why and how they changed. |
|  | Are there any social activities that you can no longer do or have begun to avoid? If so, why? |
|  | How has breathlessness affected your feelings or emotions? |
|  | What strategies or adjustments do you use yourself to cope with breathlessness? How effective are they? |
|  | What support have you received from healthcare professionals, and what support would you like to receive, to live at home with breathlessness? |
| Family caregivers | How has caring for a patient with breathlessness affected your daily life? |
|  | Are there any daily activities or schedules that you can no longer do or have changed because of caregiving? |
|  | Are there any social activities that you can no longer do or have begun to avoid? If so, why? |
|  | How has caring for a patient with breathlessness affected your feelings or emotions? |
|  | What care or adjustments do you provide for the patient’s breathlessness? How effective are they? |
|  | What support have you received from healthcare professionals, and what support would you like to receive, to care for the patient at home? |
